# Analytical Methods for Lipid Oxidation and Antioxidant Capacity in Food Systems

**DOI:** 10.3390/antiox10101587

**Published:** 2021-10-09

**Authors:** Edirisingha Dewage Nalaka Sandun Abeyrathne, Kichang Nam, Dong Uk Ahn

**Affiliations:** 1 Department of Animal Science, Uva Wellassa University, Badulla 90000, Sri Lanka; sandun@uwu.ac.lk; 2 Department of Animal Science & Technology, Sunchon National University, Suncheon 57922, Korea; kichang@scnu.ac.kr; 3 Department of Animal Science, Iowa State University, Ames, IA 50011, USA

**Keywords:** lipid oxidation, primary oxidation products, secondary oxidation products, antioxidant capacity

## Abstract

Lipid oxidation is the most crucial quality parameter in foods. Many methods were developed to determine the level of oxidation and antioxidant activity. This review compares the methods used to determine lipid oxidation and antioxidant capacity in foods. Lipid oxidation methods developed are based on the direct or indirect measurement of produced primary or secondary oxidation substances. Peroxide values and conjugated diene methods determine the primary oxidative products of lipid oxidation and are commonly used for plant oils and high-fat products. 2-Thiobarbituric acid-reactive substances and chromatographic methods are used to determine the secondary products of oxidation and are suitable for meat and meat-based products. The fluorometric and sensory analyses are indirect methods. The antioxidant capacity of additives is determined indirectly using the lipid oxidation methods mentioned above or directly based on the free-radical scavenging activity of the antioxidant compounds. Each lipid oxidation and antioxidant capacity methods use different approaches, and one method cannot be used for all foods. Therefore, selecting proper methods for specific foods is essential for accurately evaluating lipid oxidation or antioxidant capacity.

## 1. Introduction

Lipids are significant nutrients for humans and help many functional and regulatory activities in the human body, such as signal transduction, myelination, and synaptic plasticity. Lipids are also involved in the structural developments of the human body [1,2]. In food, lipid content and fatty acid composition are the two critical congenital parameters to the susceptibility of food to oxidative changes. Lipid content and the fatty acid composition of fat of farm animals varies significantly depending on animal species and the diet [3,4,5]. Fish is considered as one of the primary sources for ω-3 fatty acids, especially eicosapentaenoic acid (EPA) and docosahexaenoic acid (DHA) [6] and is highly susceptible to oxidative changes. Eggs are also rich in ω-3 fatty acids, and these fatty acids play a significant role in controlling cardiovascular diseases, inflammation, immune functions, and mental health disorders [7,8]. Not only animals but also plants contain fats and oils, which are used in cooking or preparing foods. Plant oils are rich in fatty acids, such as linoleic, linolenic, oleic, palmitic, and stearic acids, and some plant oils have industrial applications, such as coatings, inks, plasticizers, lubricants, and paints [9,10]. Plants also contain various bioactive compounds, including *p*-cymene, thymol, eugenol, carvacrol, isothiocyanate, cinnamaldehyde, cumin aldehyde, linalool, 1,8-cineol, α-pinene, α-terpineol, γ-terpinene, citral, and methyl chavicol, that are used for therapeutic, cosmetic, aromatic, fragrant, and spiritual purposes [9,11,12]. These bioactive compounds produced from plant oils are also known to have antioxidant properties [12].

**Figure 1:**
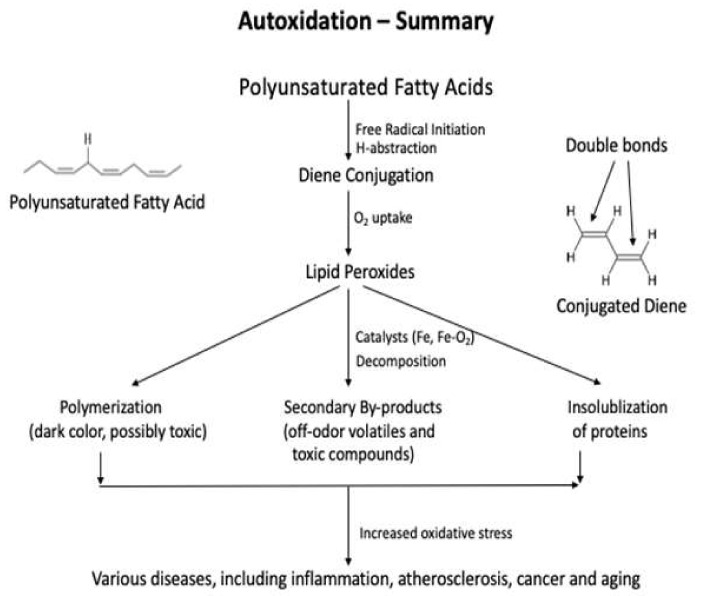
A schematic diagram of lipid oxidation pathway and the types of products produced.

Lipid oxidation causes quality deterioration in food [13,14]. Depending upon the reaction mechanisms and factors involved, lipid oxidation can be divided into autoxidation, photo-oxidation, and enzyme-catalyzed oxidation [14]. Autoxidation is the most common process of lipid oxidation in foods and is divided into initiation, propagation, and termination stages. During the initiation step, free radicals abstract the liable hydrogen atoms from the methylene group of polyunsaturated fatty acids. Then, the fatty acid rearrangement (diene conjugation) stabilizes the fatty acid radical [15,16,17,18]. In the presence of oxygen, the conjugated dienes become the peroxyl (lipid) radical with high reactivity. The total bis-allylic carbon determines the rate of peroxyl radical formation in a fatty acid molecule. Reactive oxygen species (ROS) are the most common reactive compounds that abstract a free hydrogen atom from the lipid molecules [15,18]. During the propagation, the conjugated diene becomes a highly reactive lipid radical (LOO^●^) in the presence of O_2_ and abstracts a hydrogen atom from an adjacent polyunsaturated fatty acid. Once this propagation process is started, it will continue until the termination step, where the unstable peroxyl radicals become stable non-radical [17]. The summary of the lipid oxidation is shown in Figure 1 [18].

Lipid oxidation depends on many external and internal factors. Fatty acid profile, lipid class, and fatty acid composition are the main internal factors that affect lipid oxidation. In contrast, temperature, light, moisture level, atmospheric oxygen, irons, activators, and inhibitors are the main external factors influencing food lipid oxidation and directly affecting the final product’s quality and consumer acceptance [19,20,21,22].

Since lipids play a crucial role in food quality, changes in lipids are considered one of the main parameters of the shelf-life analyses. The shelf-life of most meat and meat-based products is determined mainly by the level of oxidation, especially in foods with high lipid contents and polyunsaturated fatty acids [22,23,24]. The rancid flavor is one of the main undesirable flavor compounds produced by the lipid oxidation of raw meat and reduces the sensory qualities of the final products. Warmed-over flavors are the secondary lipid oxidation products developed in oxidized cooked meat, and aldehydes, such as hexanal, pentanal, pentanol, and nonanal compounds, are responsible for the off-flavors [18,25,26]. Some lipid oxidation products, such as malondialdehyde, acrolein, 4-hydroxy-trans-nonenal, 4-hydroxy-trans-hexanal, and crotonaldehyde-like compounds, generate not only off-flavor but also cause diseases, such as inflammation, ageing, cancer, and atherosclerosis, in humans [18,22,27]. In addition, they can interfere with the signaling pathways, leading to significant biomolecular damages [28,29]. Hydroperoxides, carbonyls, aldehydes, alcohols, furans, keto-cholesterols, epoxy-cholesterols, and oxysterols are the biomarkers produced from the enzymatic and nonenzymatic hydrolysis of lipids and are directly used to detect the physiological and pathological conditions of animals [29]. Several methods, such as antioxidants, vacuum packing, and modified atmospheric packing, have been developed to control lipid oxidation in food products [23].

Many methods are used to determine the level of oxidation in foods by detecting lipid oxidation products. The oxidation products are categorized into primary and secondary products. Critical criteria for detecting lipid oxidation include accuracy, precision, sensitivity, and detection limits [30,31]. Either direct or indirect methods determine lipid oxidation in foods. The direct methods determine lipid oxidation products, while the indirect methods use the consequences (sensory, disease conditions, etc.) of lipid oxidation or oxidation products. Peroxides and hydroperoxides are the primary oxidation compounds, and aldehydes, ketones, epoxides, alcohols, hydroxy compounds, oligomers, and polymers are the secondary compounds commonly used to determine the degree of lipid oxidation [31,32].

Antioxidants are considered a group of compounds that can counteract oxidation by acting as reducing agents, free radical scavengers, quenchers of radical species, or inactivators prooxidants, such as metals [33]. Plants contain many natural antioxidants compounds, including polyphenols (anthocyanins, flavonols, flavones), carotenoids, tannins, lignin, phenolic acids, and vitamins. However, these antioxidants can lose their activities under adverse conditions, such as high temperature, extreme pH, and strong lights [34]. The antioxidant capacity of certain compounds can be measured directly or indirectly. The direct method determines the ability of antioxidants to intervene or stop the lipid oxidation process in meat homogenates, meat products, oil emulsion, and liposome systems in the presence of antioxidant compounds. This review discusses the methods used to determine lipid oxidation and antioxidant capacity in food products and their principles and constraints.

## 2. The Analytical Methods to Determine Lipid Oxidation in Food

Lipid oxidation is considered one of the main reasons for losing the sensory and nutritional quality in many animals and plant-based foods, and various internal and external factors influence lipid oxidation in food products [18,33,35]. Methods used to determine lipid oxidation can be categorized into direct and indirect. In the direct method, the oxidation level is measured using lipid oxidation’s primary or secondary products. The most common methods determining primary oxidation products include peroxide value, iodine value, and diene conjugation measurement [32,36]. Malonaldehyde (MA), a secondary oxidation product, is the most used indicator of lipid oxidation [37,38]. The 2-thiobarbituric acid reactive substances (TBARS) methods mostly use the reaction of MA with thiobarbituric acid after distillation or acid extraction and determine the reaction products using the absorption or fluorescence spectrometer. However, HPLC and gas chromatography are also used to directly determine the amount of MA or other secondary lipid oxidation products [32,39]. The indirect measurements of lipid oxidation in foods use the fluorometric method and sensory evaluation.

### 2.1. Methods Used to Detect the Primary Oxidation Products

The three most used methods for the primary oxidation products in foods are iodometric, ferric thiocyanate, and diene conjugation methods, and they are the direct methods that determine the amount of hydroperoxides formed by oxidation in foods [40,41]. The peroxide value (PV) analysis is performed using titration or spectrophotometer, and the diene conjugation method determines all the conjugated fatty acids, including lipid peroxides, in the food products after extracting lipids from them. All three methods are used to determine the early stage of lipid oxidation, and their sensitivities are lower than those of the measurements of the secondary products. The values of the primary products can indicate the potentials of lipid oxidation at an early stage when the products are exposed to catalysts or further processed and, thus, may disagree with the sensory quality of the food.

#### 2.1.1. Peroxide Values: Iodometric and Ferric Thiocyanate Assays

The iodometric assay uses the 2H^+^ that proceeds, according to the following equations:

ROOH + 2H^+^ + 2I^−^ → ROH + H_2_O + I_2_, (1)

I^−^ + I_2_ **⇆** I_3_^−^. (2)

The liberated iodine (I_2_) is titrated with iodide Equation (1), or the reaction product of I^−^ + I_2_, triiodide anion (I_3_^−^) is measured using a spectrophotometer at 353 nm Equation (2). This method requires the extraction of lipids from the sample using Folch’s solution (chloroform: methanol = 2:1). A precaution is needed to minimize oxygen in the reaction solution because molecular oxygen interferes with the reaction and increases the background, lowering the method’s sensitivity. Thus, significant efforts should be directed to minimize oxygen in the reaction solution [42]. Under anaerobic conditions, iodometric assay displays high sensitivity and exact stoichiometry and can be carried out with minimum apparatus. Another precaution needed is eliminating any substances that promote the decomposition of hydroperoxides (e.g., transition metal ions) or that react with iodine (e.g., acetone).

The ferric thiocyanate assay also uses lipid extraction before analysis. The principle is the oxidation of ferrous iron (Fe^2+^) to ferric iron (Fe^3+^) by one-electron reduction of hydroperoxides (LOOH), followed by the homolytic cleavage of LOOH, to produce lipid alkoxyl radical (LO**^●^**), which is highly reactive, and further react with ferrous ion, solvent molecules, and LOOH Equations (3) and (4) [43].

LOOH + Fe^2+^ ---→ LO + OH^−^ + Fe^3+^, (3)

LO**^●^** + Fe^2+^ + H^+^ --→ LOH + Fe^3+^. (4)

The ferric irons (Fe^3+^) formed Equations (3) and (4) make complexes with thiocyanate, and the absorbance at 500 nm determines the amount of hydroperoxide present in the sample [44]. The final amount of Fe^2+^ oxidized depends on the nature of the solvent and the amount of LOOH present. However, the amount of ferrous iron oxidized depends on the amount of LOOH present in the sample when the solvent conditions are the same. The ferric thiocyanate method is simpler than the iodometric method, and the ferrous iron has lower sensitivity to oxygen than the iodide [45]. This method also has been successfully used to determine lipid oxidation levels in insect-based foods [46,47]. The method is easy, rapid, and sensitive and is responsive to mono- and polyunsaturated fatty acids (PUFA) hydroperoxides.

#### 2.1.2. Conjugated Diene Analysis

Autoxidation is a chain reaction in polyunsaturated fatty acids. When a hydrogen atom is abstracted from PUFA by reactive oxygen (e.g., hydroxyl radical), a double bond neighboring the oxygen-deprived carbon moves to the other double bond and forms conjugated dienes, which stabilizes the molecule [18]. In the presence of oxygen, the PUFA peroxyl radical propagates the free radical to a new PUFA, and it becomes a hydroperoxide with a conjugated form of PUFA. Thus, the analysis of conjugated diene represents the degree of oxidation at an early stage. The conjugated dienes absorb at 233 nm and, thus, can be assayed by recording the increase in the absorbance of extracted lipids at the wavelength [48]. This method is simple and determines all the conjugated forms of dienes in lipids during the early stage of lipid oxidation. The results of diene conjugation agree well with those of the peroxide and iodine values [49]. However, the diene conjugation peak is distinct at 233 nm, stands only in fully peroxidized lipids, and the peak becomes obscure in partially peroxidized lipids due to the nonperoxidized lipid and the extracted contaminants [41].

### 2.2. Direct Methods to Determine the Secondary Oxidation Products

Even though determining the oxidation level using the primary compounds is simple, large quantities of secondary (degraded) products are produced during the oxidation. Many methods are practised based on the secondary degraded products, but this review discusses the most practised methods for plant and animal-based food products.

#### 2.2.1. Thiobarbituric Acid Reactive Substances (TBARS) Method

The thiobarbituric reactive substances (TBARS) assay is one of the most popular methods to determine lipid oxidation in meat and fish products. Malonaldehyde (MA) is an aldehyde produced during the breakdown of unsaturated fatty acids [39,50,51]. One molecule of MA reacts with two molecules of thiobarbituric acid when heated in an acidic solution to form a unique Schiff base compound that gives a pink color, and the color can be measured using a visible (at 532–535 nm) or fluorescent (excitation 515 nm, emission 553 nm) spectrophotometers [50,51].

##### Absorption Spectrometry

**Figure 2:**
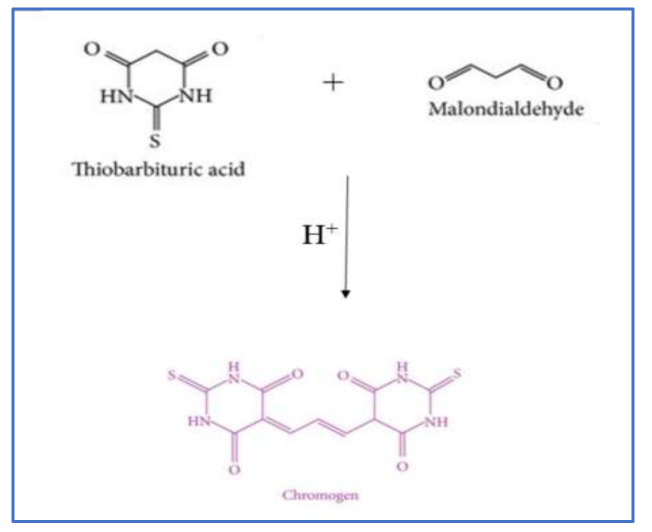
Formation of chromophore during the TBARS assay.

The chromogen formation from the reaction of thiobarbituric acid (TBA) and MA is shown in Figure 2 [52]. The reaction rate depends on the concentration of TBA, pH and the temperature used [32]. The MA can be either extracted by distillation or with an acid solution before reacting with TBA [53]. TBA is added to a sample homogenate to react with MA in the sample [41,54] directly. The distillation method is commonly used for raw, cured, and cooked meat products but is a time-consuming method. In this method, the MA is extracted with trichloroacetic acid (TCA) at a controlled rate, and the distillate is collected, reacted with TBA, and the absorbance is measured using a spectrophotometer [37]. The acid extraction is used mainly for raw and cooked meat and fish samples, which use 7.5% TCA, followed by filtration and reaction with TBA, in a boiling water bath for 30–40 min [51]. This method has also been used to determine the lipid oxidation in edible insect-based foods [46,47]. In the direct reaction method, two volumes of TBA/TCA solution (15% TCA/20 mM TBA) are added to one sample homogenate volume and are reacted at boiling temperature for 15 min. The color formed in the supernatant is collected after centrifugation, and the absorbance is read using a spectrophotometer at 532 nm. The quantification of MA in meat is performed using a standard curve and is expressed as mg of MDA/kg food [55,56]. TBARS method is easy, simple, low cost, and reproducible, and the values are well correlated with the sensory properties of the final product. However, TBA can react with other oxidized compounds in the food product and produce false-positive results [32,50,57]. The nitrite used in the cured meat products can react with MA under acidic conditions and lower value in cured meat. Therefore, the addition of sulfanilamide for both distillation and extraction methods are needed [39,50]. Production of yellow or orange chromogen is another drawback in this test and is mainly due to the reaction of sugars (sucrose, glucose, and fructose), water-soluble proteins, peptides, and free amino acids, such as Lys and Arg, in meat and meat products with the TBA. Besides the carbohydrates, phenylpropanoid-type pigments can also show absorbance at 532 nm, which result in overestimation in the oxidation [32]. The MDA can also be converted to other organic compounds with storage in fish and fish-based products, leading to a low MDA value [58]. Therefore, the TBARS method is unsuitable for determining the oxidation level in high carbohydrate-containing food materials and egg yolk.

##### Fluorometric Spectrometry

Fluorometric measurement of TBARS started from the observation of Bernheim et al. [59], who found that the oxidation of unsaturated fatty acids produced 3 carbon atoms, presumably MA, which reacted with TBA and produced a red pigment [59]. However, many constituents of food components, such as amino acids, proteins, and carbohydrates, can react with TBA [60,61]. Yagi modified the fluorometric TBARS method to determine the peroxide level in the serum or plasma using a fluorometer. He collected the water-soluble TBARS in the serum or plasma samples by precipitating proteins and lipid oxidation products under acidic conditions. Then, the precipitant was collected, suspended in distilled water, and reacted with TBA at 95 °C for 1 h. The reaction products were extracted using n-butanol, and the fluorescence intensity was measured at 553 nm with excitation at 515 nm [62]. Yagi’s fluorometric method had greater sensitivity than the spectrophotometric method but was not appropriate for meat. So, Jo and Ahn [39] further modified the method of Yagi for use in meat. They directly added TBA to the meat homogenates under acidic conditions and then reacted them at 90 °C for 15 min, extracted the reacted lipid oxidation products using the n-butanol and pyridine mixture, and then measured the intensity of fluorescence at 550 nm with 520 nm excitation. The fluorometric methods showed high sensitivity with little interference by the meat components [39]. In comparison, the fluorometric methods of Yagi and Jo and Ahn are more sensitive than the conventional spectrophotometric methods and are suitable for samples with low TBARS, such as blood serum and fresh meat samples [39,62].

#### 2.2.2. Chromatographic Methods

Because the conventional TBARS methods have insufficient specificity and sensitivity, alternative analytical approaches have been developed. The chromatographic methods are developed to determine the primary and secondary oxidation products present in the food. Kakuda et al. [63] developed an HPLC method to assess the amount of MA in aqueous distillates of chicken meat. They found that HPLC was more sensitive than spectrophotometric methods in assessing lipid oxidation products in aqueous distillates of chicken. Hydroperoxides and malonaldehyde, the primary and secondary oxidation products in foods, can also be extracted under acidic conditions first, and then the oxidation products are determined using an HPLC [64]. The method is simple, and the sensitivity is high compared with other methods but is time-consuming. Product identification and quantification can be easily made if the HPLC unit is coupled with a mass spectrometer [32,50,64,65]. Recently, a comprehensive two-dimensional liquid chromatography (LC × LC) was developed to determine lipid oxidation products using a light-scattering detector (ELSD). This method can determine both polar and non-polar lipid oxidation products. However, sample preparation for the HPLC method is complicated [65], analysis time per sample is long, and the equipment cost is high. Therefore, the industrial applications of this method are limited.

Gas chromatography equipped with mass spectrometry is another method used to determine the lipid oxidation products in foods. Specific marker compounds, such as hexanal and total aldehydes, were the most successfully used volatile compounds to determine the degree of oxidation in various foods, including meat, milk, dried, and plant-based foods, using a GC-MS [55]. In addition, the typical end products of lipid oxidation, such as aldehydes, ketones, hydrocarbons, and alcohols, that reacted with TBA are used to determine the degree of oxidation using a GC-MS [56]. Ahn et al. [66] showed that the amounts of propanal, pentanal, hexanal, 1-pentanol, and total volatiles were correlated highly (*p* < 0.01) with the TBARS values of cooked meat, but hexanal and total volatiles represented the degree of lipid oxidation better than any individual volatile compounds in cooked meat. GC method is also a sensitive method, but equipment cost is high, and analysis is time-consuming [32,66,67]. Thus, conventional spectrophotometric TBARS methods are still preferred over chromatographic methods because of their simplicity.

### 2.3. Indirect Methods Used to Detect the Secondary Oxidation Products

#### 2.3.1. Fluorometric Method

The fluorometric method is an indirect method widely used to determine the level of lipid oxidation in raw fish, meat-based products, and animal by-products, such as blood [38,58]. It can also be used on plant oils rich in polyunsaturated fatty acids (PUFA) [68,69]. In addition, the fluorometric method has been used to determine lipid oxidation in low-moisture foods, such as cereals [70]. In the fluorometric method, MA is converted to tetramethoxy-propane, which eliminates the false fluorescence readings by vitamin A, nicotinamide adenine dinucleotide (NADH), proteins, and amino acids. Thus, this method is more suitable for protein-rich foods with more ionic and covalent bonds, such as fish [71]. The most common method includes lipid extraction using a chloroform-methanol-water solvent. The collected organic layer is mixed with sodium sulfate and filtered. Then the mixture is dried with nitrogen gas at a temperature not exceeding 35 °C. The MA equivalent in lipid extracts is measured by reacting with TBA. BHT is added to the extraction solvent, and the secondary reaction products of lipid oxidation are measured by fluorescence excitation (360 nm) and emission (440 nm) of the aqueous and organic layers that contain chloroform and methanol (2:1) [68,69]. The fluorescence produced is reliable and sensitive to detecting lipid and water-soluble hydroperoxides in meat and fish samples [72]. The sensitivity of this method is higher than the absorption spectrophotometric methods (TBARS method) and is reproducible, simple, and needs a tiny amount of sample to detect the level of oxidation in meat and meat-based products.

Lipofuscin is commonly used as a fluorescent marker, but some other markers, such as 1,4-dihydropyridines and 1-palmitoyl-2-((2-(4-(6-phenyltrans-1,3,5-hexatrienyl)-phenyl)-ethyl)-carbonyl)-sn-glycero-3-phosphocholine (DPH-PC), diphenyl-1-pyrenylphosphine (DPPP), are also used as a new type of fluorescent compounds in food. These markers are dissolved in a chloroform/ methanol mixture, and the solvents are removed using nitrogen. The dried sample is mixed with phosphate buffer, and the reaction progress is observed at 430 nm (excitation at 354 nm) at 37 °C using a fluorimeter [72]. With these markers, image processing is introduced as a novel method to determine the level of oxidation in foods [71,72].

#### 2.3.2. Sensory Analysis

Sensory evaluation evokes, measures, analyzes, and interprets human responses to the properties of food materials. A trained or untrained panelist can determine the sensory attributes, such as smell, flavor, and taste of the food product [73]. Sensory evaluation is considered a critical method since it directly interprets oxidation levels in a food product [74,75,76]. In a practical situation, this evaluation is performed by field panels, consumer panels, free choice profiling panels, quantitative description analysis panels, and expert panels, especially with low-moisture foods, such as cereals [70,73,77]. Sensory evaluation is cost-effective and simple and can be applied to all plant- and animal-based foods in liquid, semi-solid, and solid forms. However, the sensory analysis shows high regional variations and low repeatability because of the personal preference and experience, the cultural backgrounds, the age and sex of panelists, and the time of the evaluation performed. Therefore, a sensory, along with the chemical and instrumental, analysis is recommended. As discussed above, many methods are available to determine the degree of lipid oxidation in food, but one method cannot fit all food products. Thus, selecting a proper and effective method for a specific food is essential.

### 2.4. Methods Used to Detect the Primary and Secondary Oxidation Products

*p*-Anisidine test (p-AV) and total oxidation index (TOTOX) are two common methods used in determining lipid oxidation in food products, especially edible oils. Both *p*-anisidine and TOTOX tests determine both the primary and secondary oxidation products in oil [78]. However, the anisidine test uses aldehydes, such as 2-alkenals and 2,4-alkadineals, as the main oxidation markers, and the level of anisidine produced is measured using the UV-spectrophotometer at 350 nm [78,79].

TOTOX indicates the overall oxidation states, and it can give a good explanation of the final quality of the edible oil. It is calculated using the following equation:

TOTOX= *p*-Anisidine value + 2 Peroxide value.

Both *p*-AV and TOTOX methods are simple, require less technical knowledge, and are commonly used to determine the quality of the EPA/DHA-containing oils and finished products [79,80]. However, *p*-AV is not suitable for determining the oxidation in omega-3-rich oils with intense color or specific flavorings added to the final oil [80]. Table 1 shows the summary of lipid oxidation methods with their principles, applications, advantage, and disadvantages of each method.

## 3. Methods for Antioxidant Capacity Measurement

### 3.1. Direct Measurement of Antioxidants

The direct measurement of antioxidants uses model systems and determines the antioxidant capacity using DPPH radical scavenging assay, ABTS radical scavenging assay, total phenolic content analysis ferric reducing antioxidant power (FRAP) assay, CUPRAC assay, and electrochemical methods.

#### 3.1.1. 2,2-Diphenyl-1-Picrylhydrazl (DPPH) Radical Scavenging Assay

**Figure 3:**
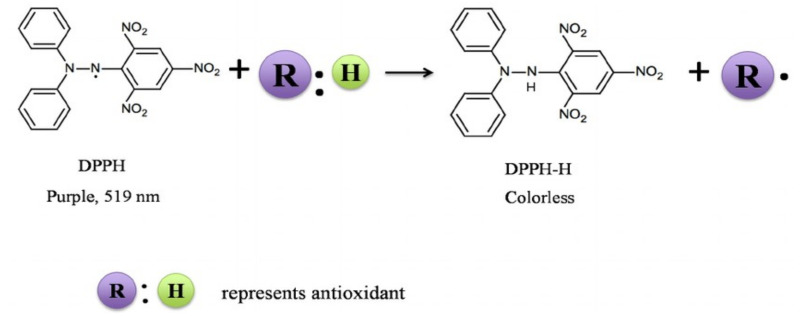
Changes in the DPPH radical-scavenging activity.

The DPPH radical scavenging assay is a spectrophotometric method that determines an antioxidant molecule’s free radical scavenging potential. It is considered one of the standard methods to determine the antioxidant property [81]. The DPPH method is mainly used to determine the potentials of antioxidants in plants, seaweeds, herbs, edible seeds, plant oils, cereals, honey, and flours [82,83,84,85]. DPPH is a stable free radical and forms a characteristic purple color. During the scavenging of the free radicals in the sample, these colored compounds will turn to the colorless hydrazine and be measured at 515–528 nm (Figure 3) [86].

There are many variations, mainly the concentration of DPPH solution (22.5–250 µM) and the incubation time (5–60 min), among the methods used to determine the DPPH scavenging assay [87]. The level of radical scavenging ability is expressed in ascorbic acid equivalent. During the reaction, the initial electron transfer occurs very quickly, but hydrogen transfer takes time. This rate depends on the solvent (ethanol or methanol) used on the acceptance of H-bonds. However, the stability of DPPH^●^ is higher in methanol than ethanol; thus, methanol is commonly used as a solvent in DPPH assay [83].

#### 3.1.2. 2,2′-Azino-Bis-3-Ethylbenzothiazoline-6-Sulfonic Acid (ABTS) Radical Scavenging Assay

**Figure 4:**
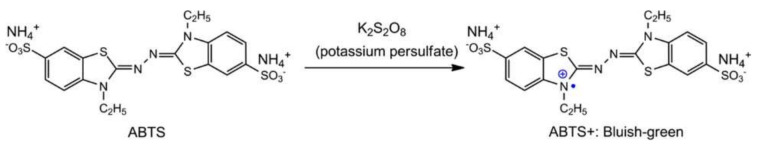
Changes in the ABTS radicals with potassium persulfate.

The ABTS assay is another method used to determine the antioxidant activities based on the free radical scavenging. The most common method used to determine the ABTS radical scavenging assay described by Re et al. [88], and the chemical changes during the oxidation are shown in Figure 4 [89]. In this method, the ABTS^●+^ cation radical reacts with myoglobin, hydrogen peroxide, or other organic compounds present in plants (e.g., perborate and luminol). The reactive oxygen and nitrogen species (ROS/RNS) significantly affect the final antioxidant property of foods. The production of ROS and RNS determines the presence of enzymes (superoxide dismutase, catalase, glutathione peroxides), metal ions, and chemical compounds, such as ascorbic acids, tocopherols, uric acid, and flavonoids [89]. This method is more suitable for samples containing multiple ingredients and has complex reaction kinetics [90]. Since ABTS^●+^ can be dissolved in both water and inorganic media, this method can determine the oxidation level in water-soluble and water-insoluble foods. It is also a simple method in which data were collected using a UV-VIS spectrophotometer [89]. The absorbances are read at 414, 752, and 832 nm for water-soluble samples, while 414, 730, and 873 nm are used in ethanol media [90]. The main limitation is reaching the endpoint after reacting the sample with the ABTS^●+^ [91]. In addition, the ABTS radicals used in the assays are not found in a typical biological system [87]. Therefore, the interpretation of the oxidation in the food system may not be accurate. The ABTS method is used widely to determine the antioxidant activity of plant and plant-based products [84,87] but is also proposed to check the antioxidant potential of a compound as a therapeutic efficacy in human tissues based on the potential reaction of the free radicals, such as ^●^OH or O_2_^•−^ with ABTS [92,93].

#### 3.1.3. Ferric Reducing Antioxidant Power (FRAP) Assay

**Figure 5:**
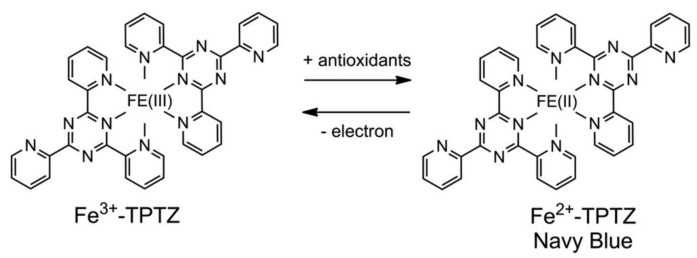
Formation of Fe^3+^ and Fe^2+^ during the electron reception and donation in FRAP assay.

Reducing power involves electron-accepting and donating the capacity of food components. The principle of the FRAP method is the conversion of Fe^3+^ to Fe^2+^ by antioxidants [94,95]. Fe^2+^ binds with the ligands forming a dark blue color during the reaction, determined using a UV-spectrophotometer at 593 nm. The results can be expressed ad micromolar equivalents of Fe^2+^ or using a standard curve (Figure 5) [85,95]. The following reactions illustrate the reactions between Fe^2+^ and antioxidants in the food [95].

Antioxidant + Fe^3+^ ⇆ Fe^2+^ + oxidized antioxidant,

Fe^2+^ + Fe(CN)_6_^3−^ ⇆ Fe[Fe(CN)_6_]^-^.

This method is fast, simple, and cheap. In addition, it does not require high-tech equipment to detect oxidation [95]. However, the results can vary with the analysis time and the pH of the reaction media [94,95].

#### 3.1.4. Cupric Reducing Antioxidant Capacity (CUPRAC) Assay

**Figure 6:**
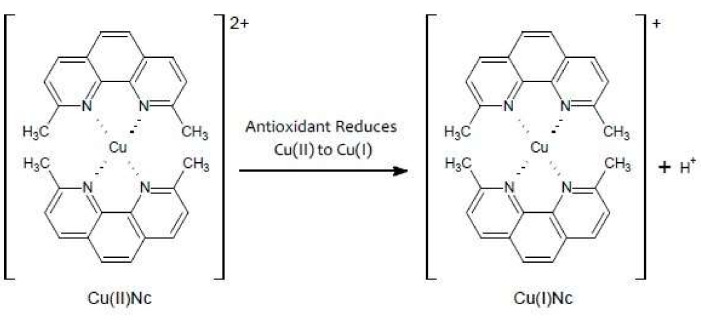
Chemical change in CUPRAC assay.

The principle of CUPRAC assay is similar to that of the FRAP assay, where the Cu^2+^ converts to Cu^+^ during the electron transfer by various polyphenols (flavonoids, carotenoids, vitamin C and E, phenolic acids). The chemical widely used in this assay is bis-copper (II) cation, which acts as an outer-sphere electron transferring agent. The change of a chemical reaction is shown in Figure 6 [96]. The cupric reagent is reduced during the reaction, and the color changes from light blue to yellow-orange [79,94]. The CUPRAC assay is also a cost-effective and simple method that does not require high-tech equipment.

#### 3.1.5. β-Carotene Bleaching Assay

**Figure 7:**
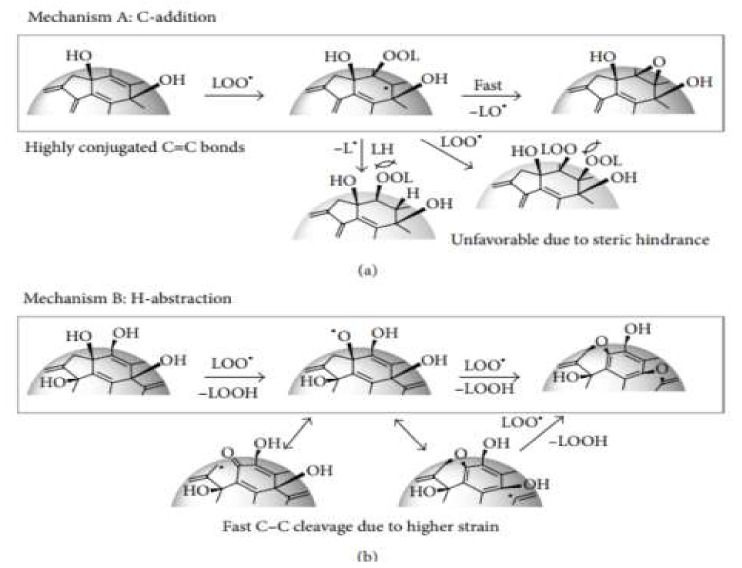
Possible mechanism of β-Carotene bleaching assay. (**a**) Mechanism A: C-addition, (**b**) Mechanism B: H-abstraction.

It is one of the oldest methods to quantify antioxidant and antioxidant activity [87,96,97]. This method also consumes free radicals and behaves similarly to DPPH, ABTS, FRAP, and CUPRAC assays [82,98,99,100]. During this method, discoloration occurs when the β-carotene reacts with the free radicals in the sample, which is determined using the UV-spectrophotometric at 470 nm [82,100]. The mechanism is the discoloration of the yellowish color of a β-carotene solution due to the breaking of *π*-conjugation by the addition reaction of free radicals to a C=C double bond of *β*-carotene, as shown in Figure 7 [101].

Although the β-carotene bleaching assay is widely used in oils, plant, and plant-based products, this method has low reproducibility and is sensitive to oxygen concentration and temperature, even when linoleic acid is absent [97]. The solvent types used, the ratio of the solvent volume to β-carotene, and the pH of the sample also influence the assay [96].

#### 3.1.6. Total Phenolic Content (TPC) Analysis

Total phenolic content usually determines the level of antioxidants in plants and plant-based products. Most plant materials show antioxidant properties because of the phenolic substances, the secondary metabolites, in the plant [94,101,102]. Solvent extraction is mainly used to obtain phenolic compounds, and ethanol, methanol, and acetone are the common organic solvents used [103]. The activity of compounds mainly depends on the pH of solvents, the polarity of the compounds, temperature, and the chemical structure of the organic compound. The UV-spectrophotometer is commonly used to detect the TPC, but high-performance liquid chromatography (HPLC), gas chromatography (GC), and capillary zone electrophoresis (CZE) are also used [101]. Because each phenolic compounds have different antioxidant potency, TPC cannot represent the actual capacity of the antioxidant in a product.

#### 3.1.7. Electrochemical Methods

The electrochemical method is a new technology used to determine the antioxidant activity in food products. It reduces the drawbacks of spectrophotometric methods, such as expensive chemicals, undefined reaction time, low precision, low sensitivity, and long sample preparation time. The oxidative stress of food and living organisms is measured using electrochemical methods [104,105], including cyclic voltammetry, differential pulse voltammetry, square wave voltammetry, and chronoamperometry [105]. The cyclic voltammetry method provides a wide range of information, such as the thermodynamics of the redox process and kinetics of heterogeneous electron transfer reactions [104]. The differential pulse voltammetry also uses the same principle as the cyclic voltammetry method. The square-wave voltammetry is widely used with very high sensitivity when net current is higher than either forward or reverse components [105]. The electrochemical methods are developed, along with the functional foods and their importance, and can determine the low molecular weight antioxidant compounds present in food. The electrochemical methods are quick, simple, cheap, and minimize interference from other compounds on the final reading. In addition, they have less interference by the pH of the solvents, solvent polarity, and concentrations [106]. Other than that, the electrochemical method allows a high number of parameters to be obtained in a single experiment [104,105]. However, applications are limited due to the electrochemical sensors used. Chemicals, such as NaCl or KCl, are mixed with the solvents and used for electrochemical analysis. These ions can interfere with the oxidation process. In addition, some antioxidants, such as polyphenols, present in food can produce a passivating film that can reduce the conductivity of the electrodes used [106]. The electrochemical methods show similar results to DPPH, FRAP, and ABTS radical scavenging activities; therefore, the results’ accuracy is high [107]. The electrochemical methods are widely used in plant-based products [107,108] and less applied in animal-based products.

### 3.2. Indirect Measurement of Antioxidant Capacity

The indirect methods determine the effect of an antioxidant on the prevention of oxidation in foods using TBARS, fluorometric, peroxide value, diene conjugation, iodine value, and HPLC and GC methods, as discussed above. The indirect methods use meat homogenates, meat products, oil emulsion, and liposome systems, and the results of antioxidant capacity obtained by the indirect methods often do not agree with those of the direct assays, especially in meat systems. Thus, the indirect measurement of antioxidant capacity is recommended for antioxidants’ practical and tangible effect in meat-based food systems.

### 3.3. Other Common Methods Used to Measure Antioxidant Capacity

Other than the methods mentioned above, oxygen radical absorption capacity (ORAC), hydroxyl radical antioxidant capacity (HORAC), and total peroxyl radical trapping antioxidant parameter (TRAP) are also used to determine the antioxidant strength of foods.

The ORAC assay measures the free radical chain-breaking ability of antioxidants by monitoring the inhibition of peroxyl radicals. The peroxyl radicals are the main free radicals produced during lipid oxidation in foods and other biological systems. Therefore, measuring peroxyl radicals under fluorescence is practised. The Trolox, a water-soluble analogue of vitamin E, is used as the standard reference, and the value is expressed as Trolox equivalent [79,109]. The ORAC assay is a hydrogen atom transfer reaction-based assay, and it measures the radical chain-breaking capacity of antioxidants. The ORAC method monitors antioxidants’ free-radical inhibiting capacity by monitoring the decrease of fluorescence signal from a probe by reactive oxygen species [79]. The most common probes used in the ORAC assay include α,α-azobisisobutyronitrile, 2,2-azobis(2-amidinopropane) hydrochloride and AMVN (2,2′-azobis(2,4-dimethylnaleronitrile) (ABAP), and the hydrophilic 2,2′-azobis(2-amidinopropane) dihydrochloride (AAPH) [110]. The fluorescent B-phycoerythrin (B-PE), a protein isolated from a species of red algae (Porphyridium cruentum), is also used in the ORAC assay, but this compound has nonspecific binding sites and gives false interpretation to the final value [111]. Alternatives are being introduced to overcome this problem, and they showed positive results with fruit juices and wine and gave accurate, precise values [112].

The main principle behind the HORAC assay also uses the hydrogen atom transfer mechanism and evaluates the capacity of antioxidants in inhibiting the oxidation of fluorescein by hydroxyl radicals. This method uses hydrogen peroxide to generate hydroxyl radicals, which quench the fluorescence of fluorescein (excitation at 485 nm and emission at 520 nm) until all the antioxidant activity in the sample is depleted. After that, the hydroxyl radicals react with the fluorescein and quench the fluorescence. The area under the fluorescence decay curve is used to quantify the total HORAC in the sample, calculated using the standard curve prepared by gallic acid (GA). The value is expressed as the GA equivalent, and the assay directly measures the antioxidant capacity against hydrophilic chain-breaking hydroxyl radicals [113,114].

The TRAP is another method that determines the total free-radical trapping capacity of antioxidants in foods. The principle of this method is determining the peroxyl radicals produced during thermal decomposition are reacted with the luminol-enhanced chemiluminescence. The TRAP assay generates peroxyl radicals using 2,2′-azobis (2-amidinopropane) dihydrochloride (AAPH), and the oxidation reactions are monitored by oxygen consumption using an oxygen electrode. The idea is that the length of the lag phase before increased oxygen consumption is proportional to the antioxidant capacity of the sample. The TRAP values calculated from the length of the lag-phase caused by the antioxidant capacity are expressed as the Trolox equivalents [109,113,115]. The summary of the methods used to determine the antioxidant capacity in foods is listed in Table 2.

## 4. Summary

Lipid oxidation is a crucial quality problem in many processed foods. Therefore, determining the level of lipid oxidation is essential to monitor the quality and assure consumer acceptability of foods. Numerous methods have been developed to determine lipid oxidation in foods and biological materials, and each of the methods uses different approaches, indicators, or targets for the analysis. Food products have different compositional and physicochemical characteristics and the stage of lipid oxidation at the time of measurement. Therefore, some methods can work better than others, depending on the food, oxidation stage, and lipid oxidation level. For more accurate information about the oxidation status and antioxidants’ capacity, selecting the most appropriate analytical methods for each food product or antioxidant would be very important. Among the many methods practised in determining lipid oxidation, peroxide, iodine values, and diene conjugate are the direct methods used for detecting the primary substances produced by oxidation. These methods are practical for oils, high-fat foods, and raw meat products, and their sensitivity is generally low. The TBARS and chromatographic methods determine the levels of secondary products of lipid oxidation. The TBARS assays are the most widely used method to determine lipid oxidation in animal-based foods and are also simple and effective. However, TBARS methods can have a low expression level in carbohydrate-rich products because MA can react with sugar, generate a yellowish color instead of pink, and are unsuitable for oils or phospholipids-rich products. Fluorometric method and sensory evaluation are indirect detection methods of lipid oxidation in food products. Sensory evaluation is a suitable method, but its repeatability is low, and person to person variations cannot be controlled. *p*-Anisidine and TOTOX methods determine primary and secondary oxidation products in foods and are suitable for edible oils.

Antioxidant capacity in food decides the storage stability of foods and can be determined by direct and indirect methods. The direct measurement of antioxidant capacities, such as ABTS, TPC, DPPH, FRAP, CUPRAC, and β-carotene methods, are widely used in plant-based foods, and they are simple and effective. The indirect methods for antioxidant capacity measurement include TBARS assay, peroxide value, iodine value, and conjugated diene methods. Methods, such as ORAC, HORAC, and TRAP methods, use indirect calculation for antioxidant capacity. Although the antioxidant capacity can be measured either by direct or indirect methods, they sometimes do not agree well on the food products. The analysis of lipid oxidation and antioxidant capacity using multiple methods sharing similar principles would not improve the accurate evaluation of oxidation status nor the antioxidant potential of antioxidant compounds or foods. Thus, selecting a suitable method for a specific food or using one or two methods with different mechanisms will be more economical and informative in evaluating the degree of oxidation or antioxidant capacity than using several analytical methods with similar analytical principles.

## Figures and Tables

**Table 1:** Summary of the common methods used in lipid oxidation.

Lipid Oxidation Analysis Method	Principle of the Method	Possible Applications	Advantages of the Method	Disadvantages of the Method	References
Peroxide value (PV): Iodometric and ferric thiocyanate	Oxidation of iodide by hydroperoxides or by oxidation of Fe^2+^ to Fe^3+^. Use a spectrophotometer to obtain the final reading.	Plant oils and liquid food products, edible insects.	Simple and cheap. Direct readings. Under anaerobic conditions, the sensitivity is high.	Depend on the titration skills of the person. Only applicable to liquid-based products.	[42,43,44,45,46,47]
Conjugated diene analysis	Isomeric hydroperoxides will make conjugated dienes with the removal of oxygen and determine 1,4-dienes produced. Measured at 233 nm.	Suitable for PUFA-containing foods.	Gives actual values of LDL oxidation during the early stages of oxidation. Simple and cheap.	Depend on the composition and size of the lipoproteins. Small, conjugated dienes are difficult to detect.	[18,41,48,49]
TBARS assay	Detect the production of chromogen due to the reaction of MA and TBA. Read absorption at 532 nm. HPLC, GC-MS, and fluorometer are also used.	Meat and meat-based products. Fish and fish-based products. Can be used in cured meat products, edible insects.	Simple and fast detection. Easy to detect and low cost. Reproducible and correlate well with sensory attributes.	MA and TBA can react with other organic compounds present in food. Absorption spectrophotometer is not suitable for detection at low levels.	[32,37,39,50,51,52,53,54,55,56,57,58]
Chromatography methods	By using the HPLC or GC. Determine the specific compounds produced.	All types of raw and processed foods. Oxidative stress-related diseases.	Sensitive and accurate. Identification and quantification can be made.	The cost of the equipment is high. The complexity of the method and the fact that it is time-consuming.	[32,50,55,56,64,65,66,67]
Fluorometric method	Use different fluorescent porphyrins to interact with MDA produced during oxidation.	Animal-based products. Can be used to determine the changes in human serum/plasma. Low moisture foods.	Fast and accurate. Non-destructive Sensitive. Image produced can be further used. Can be used to detect unstable oxidized compounds.	High cost of the equipment used. Complexity of the method.	[38,58,68,69,70,71,72]
Sensory analysis	Use trained or untrained human panelist to determine the level of oxidation through sensory attributes, such as odor, taste, and acceptability.	All animals and plant-based foods, cereals.	Gives the overall quality of the food. Direct interpretation. Can be used for liquid, semi-solid, and solid foods.	Depends on the individual participants and time variations. Depends on the region. Reproducibility difficult. Ethical clearance is needed.	[73,74,75,76,77]
*p*-Anisidine test	Determine the level of anisidine produced from the secondary aldehydes produced.	Oil and oil-based products.	Simple, less technical knowledge needed.	Problems in omega-3-rich oils that contain intense colors or containing specific flavorings.	[78,79,80]
Total oxidation index (TOTOX)	Determine the total oxidizied products.	Oil and oil-based products.	Simple calculation.	Similar problem associated with *p*-anisidine test.	[78,79,80]

**Table 2:** Summary of the methods used to measure the antioxidant capacity of foods.

Antioxidant Method	Principle	Uses/Applications	Advantages	Disadvantages	References
DPPH assay	Determine the free radicals produced during the oxidation. Reduction of purple color is measured using a spectrophotometer (515–528 nm).	Can be used to detect the oxidation in plants, seaweeds, herbs, edible seed, and plant oils.	Simple, fast, and Cheap.	Depends on the solvent used. The presence of particles will interfere with the results.	[81,82,83,84,85,86,87]
ABTS assay	Determine the free radicals produced during the oxidation and reduction.	Plant and plant-based products.	Water-soluble and insoluble compounds can be analyzed. Simple and fast.	Depends on the enzymes, metal ions, energy provided, and chemicals in the food. Not suitable in biological systems.	[84,88,89,90,91,92,93]
FRAP assay	Reducing power involves electron-accepting and donating Fe^3+^ → Fe^2+^.	Plants and plant-based products.	Simple, fast, and cheap. No enzyme involvement.	Varies with analyzing time.	[94,95,96]
CUPRAC assay	Reducing power involves electron-accepting and donating Cu^2+^ → Cu^+^.	Plants and plant-based products.	Simple and cost-effective. No need for high-tech equipment.	Not suitable to combine with TPC, ABTS, FRAP, or DPPH assays.	[79,94,96]
β-carotene assay	Determine the free radicals produced during the oxidation and reduction.	Plants and plant-based products. Plant oils.	Simple and fast.	Sensitive to oxygen and temperature when linoleic acid is absent. Low reproducibility. Depend on solvent type, ratio, pH of the sample.	[82,87,96,97,98,99,100,101]
TPC assay	Determine the levels of antioxidants.	Plants and plant-based products.	Simple, fast, and Cheap.	Activity depends on pH, the polarity of solvent and temperature. Depends on external factors.	[94,101,102,103]
Electrochemical assay	Based on thermodynamics of the redox process and kinetics of heterogeneous electron-transfer reactions.	Animal-based and plant-based products. Functional foods.	Reduce the problems in spectrophotometer methods. Short sample preparation time. Low reaction time. Sensitive and reduce the interference.	Cost of the equipment used. Complexity of the method. Some polyphenols can produce passivating films. Chemicals, such as NaCl/KCl, interfere with the electrolytes.	[104,105,106,107,108]
Oxygen radical absorption capacity (ORAC)	Based on the breaking of peroxyl radical chains reaction by antioxidants. Monitor the inhibition of peroxyl radicals.	Biological fluids and functional foods. Plants and plant-based liquid products.	Accurate, precise values can be obtained. Can be used to detect both hydrophilic and hydrophobic antioxidants.	Can easily bind with other compounds and give false values. Technically demanding method.	[79,109,110,111,112]
Hydroxyl radical antioxidant capacity (HORAC)	Based on the breaking of hydroxyl radical chains reaction by antioxidants.	Biological fluids and functional foods. Plants and plant-based liquid products.	Direct measurement of antioxidant capacity. Sensitive.	Time dependent. Indirect calculation, technically demanding.	[113,114]
Total peroxyl radical trapping antioxidant parameter (TRAP)	Based on the quenching of chemiluminescence.	Used in plasma and cerebrospinal fluids.	Suitable for liquid foods.	Time-dependent. Indirect calculation. Complex technology is needed. Comparisons between labs are difficult.	[109,113,115]
